# Supplementary figures and images for: Excretory/Secretory Products from Schistosoma japonicum Eggs Alleviate Ovalbumin-Induced Allergic Airway Inflammation
Source: PLoS Negl Trop Dis. 2023 Oct 3;17(10):e0011625. doi: 10.1371/journal.pntd.0011625 (PMC10547495; doi:10.1371/journal.pntd.0011625)

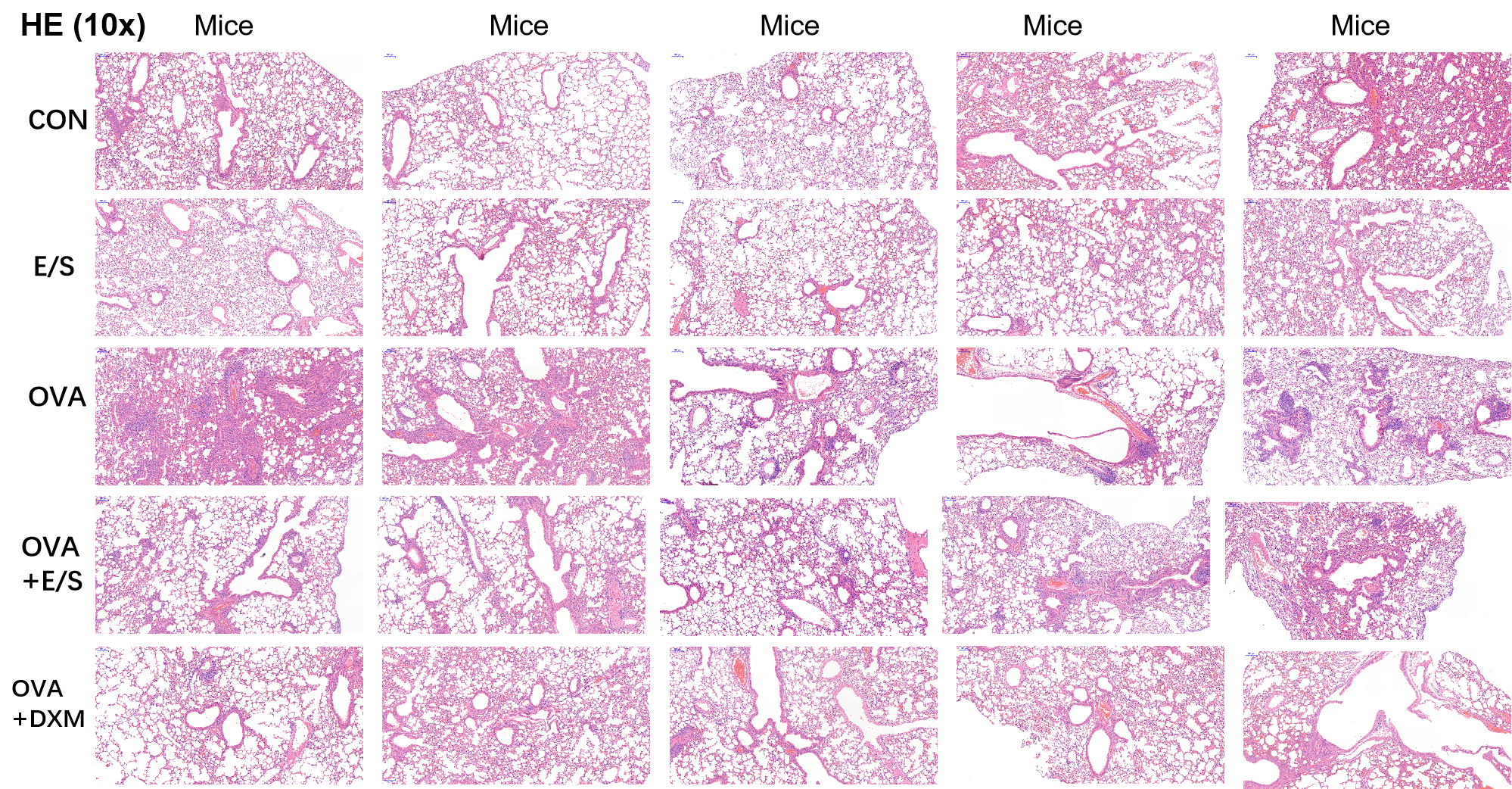

Supplement: S1 Fig — CON, normal mice (without OVA sensitization and challenge); E/S, mice without OVA sensitization and challenge but treated with ESP-SJE; OVA, mice with OVA sensitization and challenge but without ESP-SJE treatment; OVA + E/S, mice sensitized and challenged with OVA and treated with ESP-SJE; OVA + DXM, mice sensitized and challenged with OVA and treated with dexamethasone. (TIF) [file pntd.0011625.s002.tif]

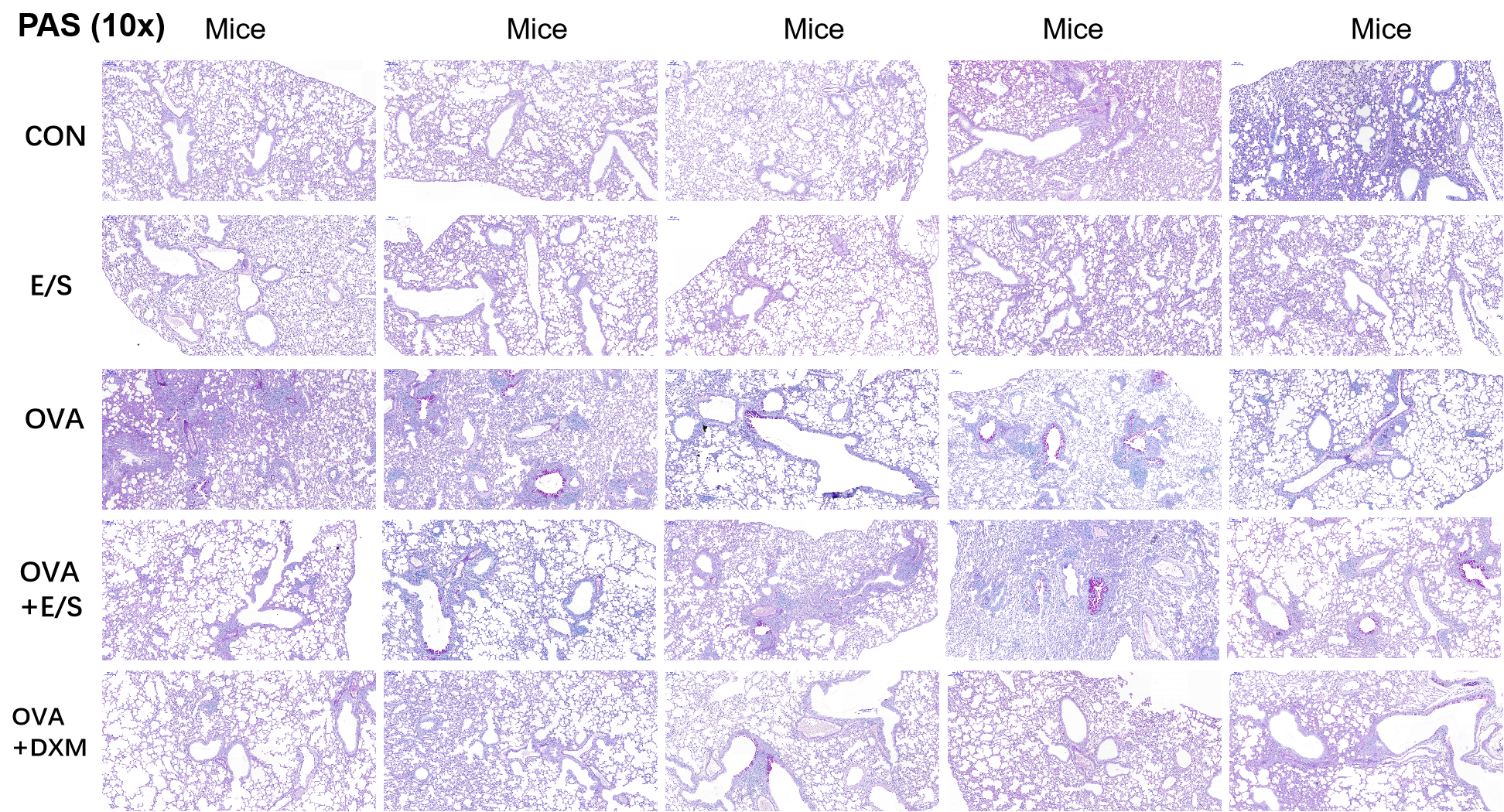

Supplement: S2 Fig — (TIF) [file pntd.0011625.s003.tif]

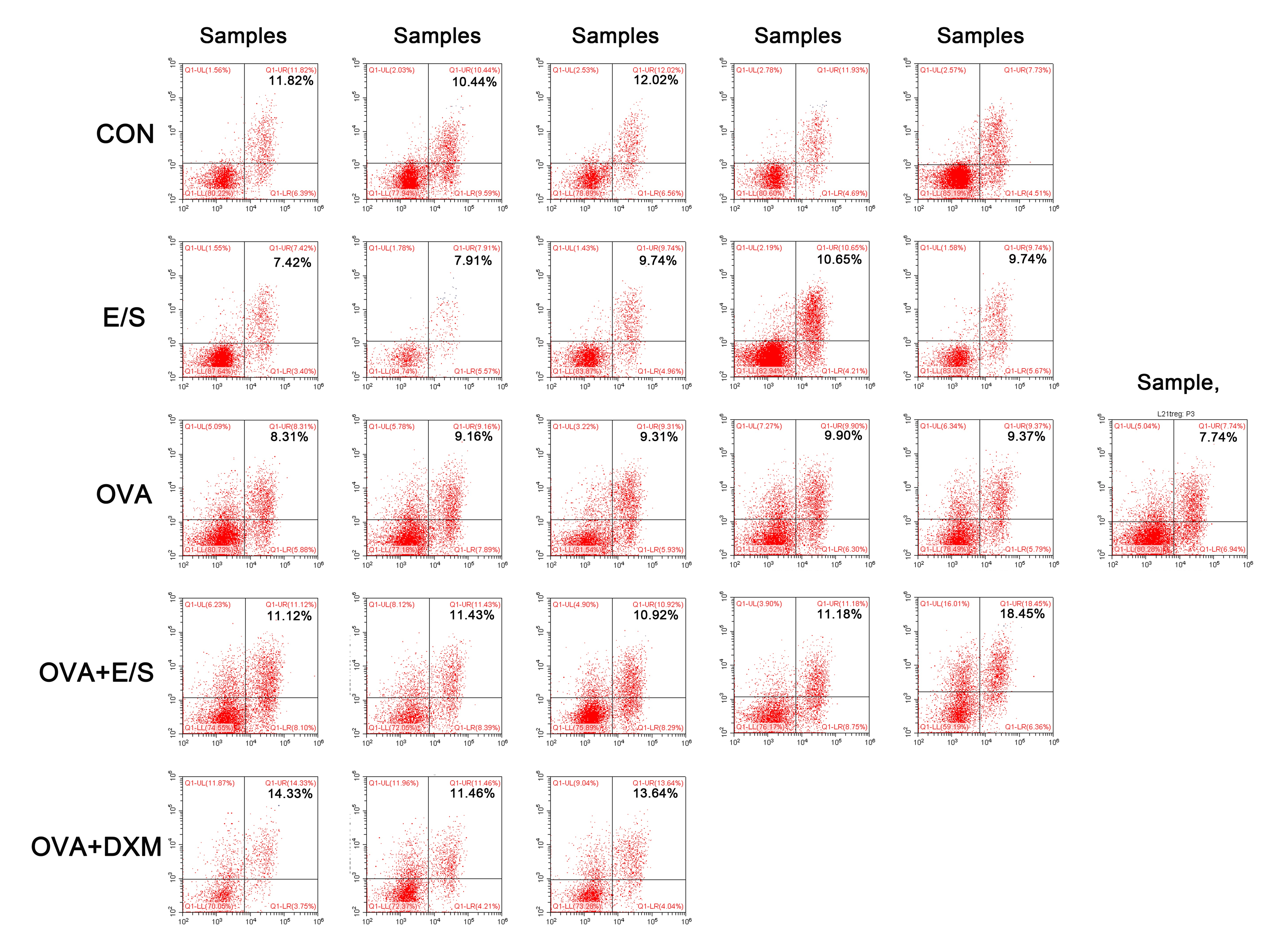

Supplement: S3 Fig — (TIF) [file pntd.0011625.s004.tif]

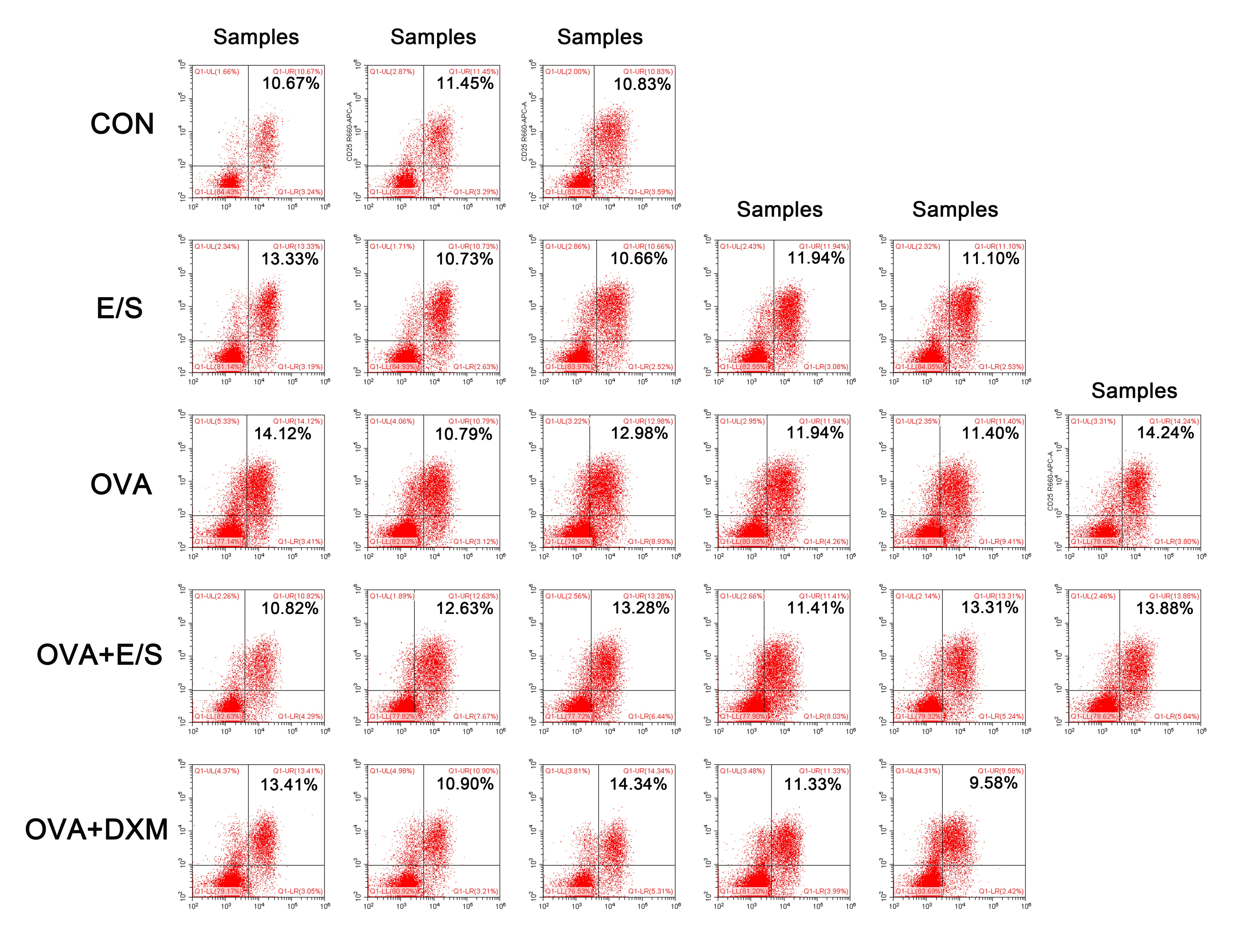

Supplement: S4 Fig — (TIF) [file pntd.0011625.s005.tif]

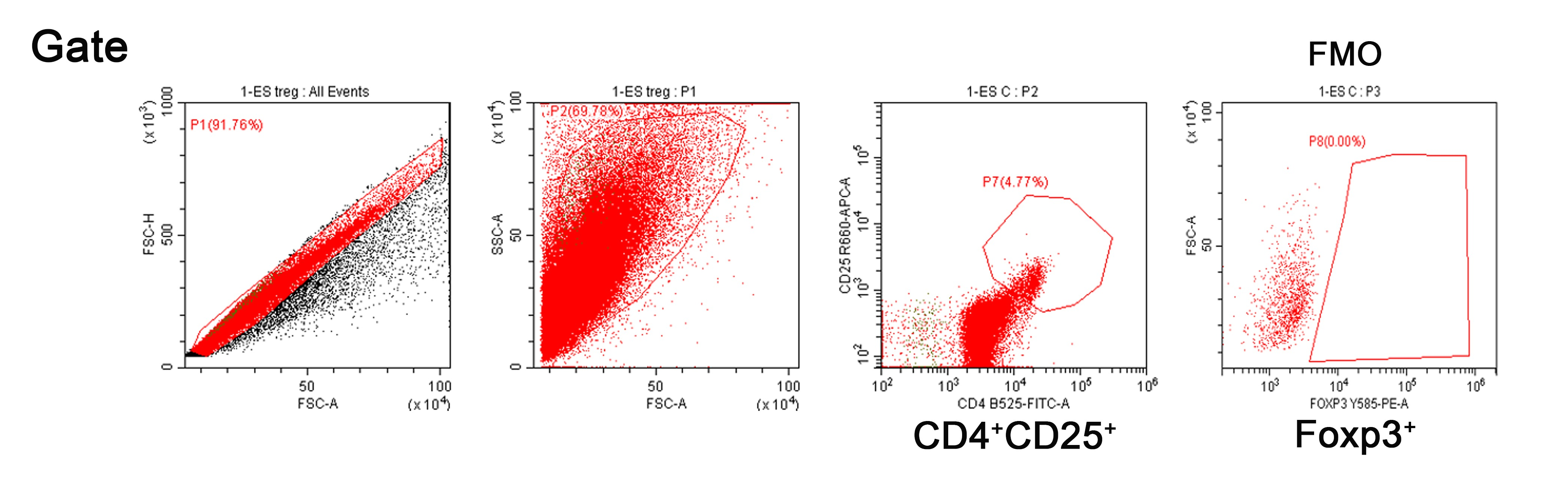

Supplement: S5 Fig — (TIF) [file pntd.0011625.s006.tif]

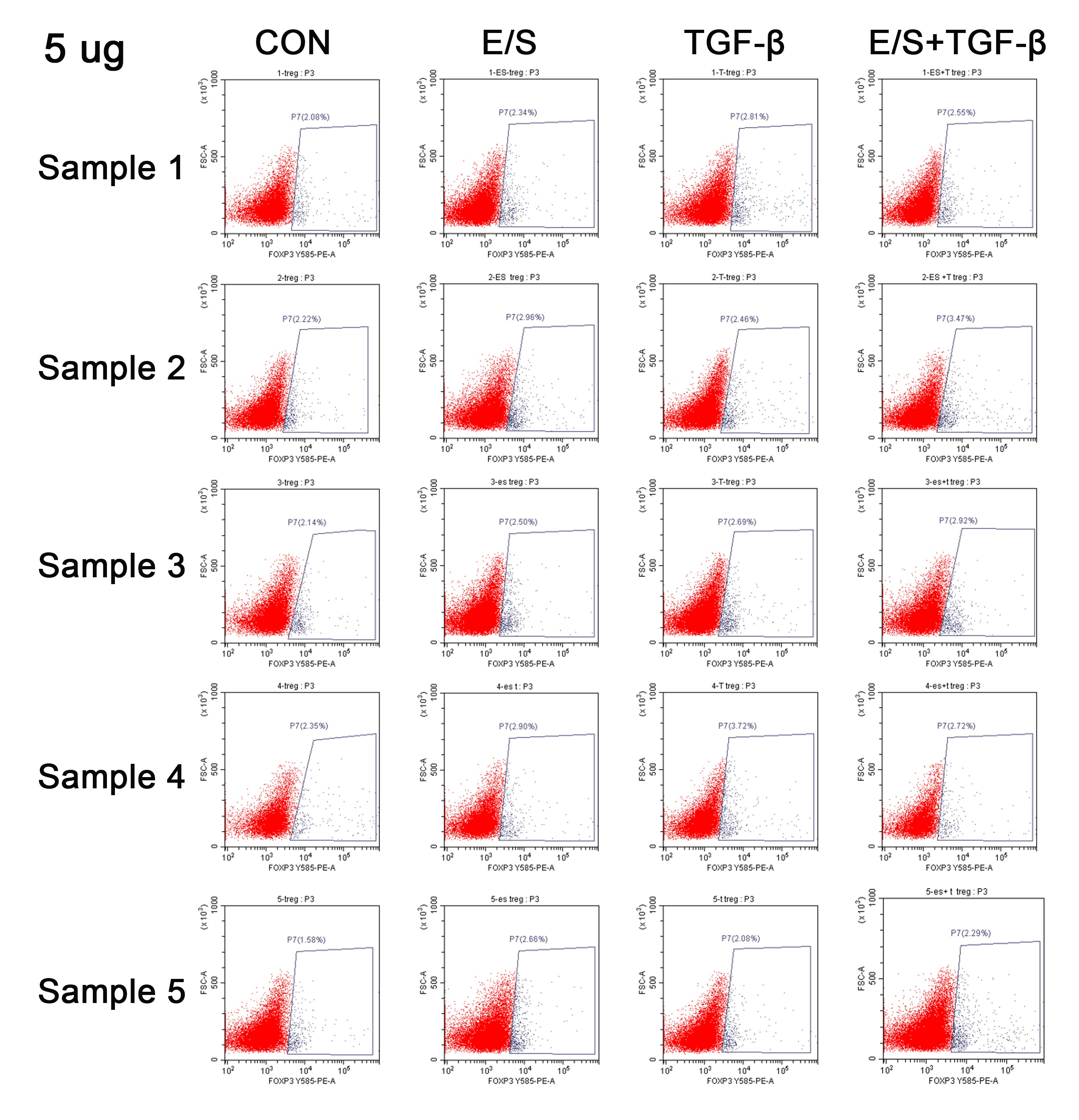

Supplement: S6 Fig — (TIF) [file pntd.0011625.s007.tif]

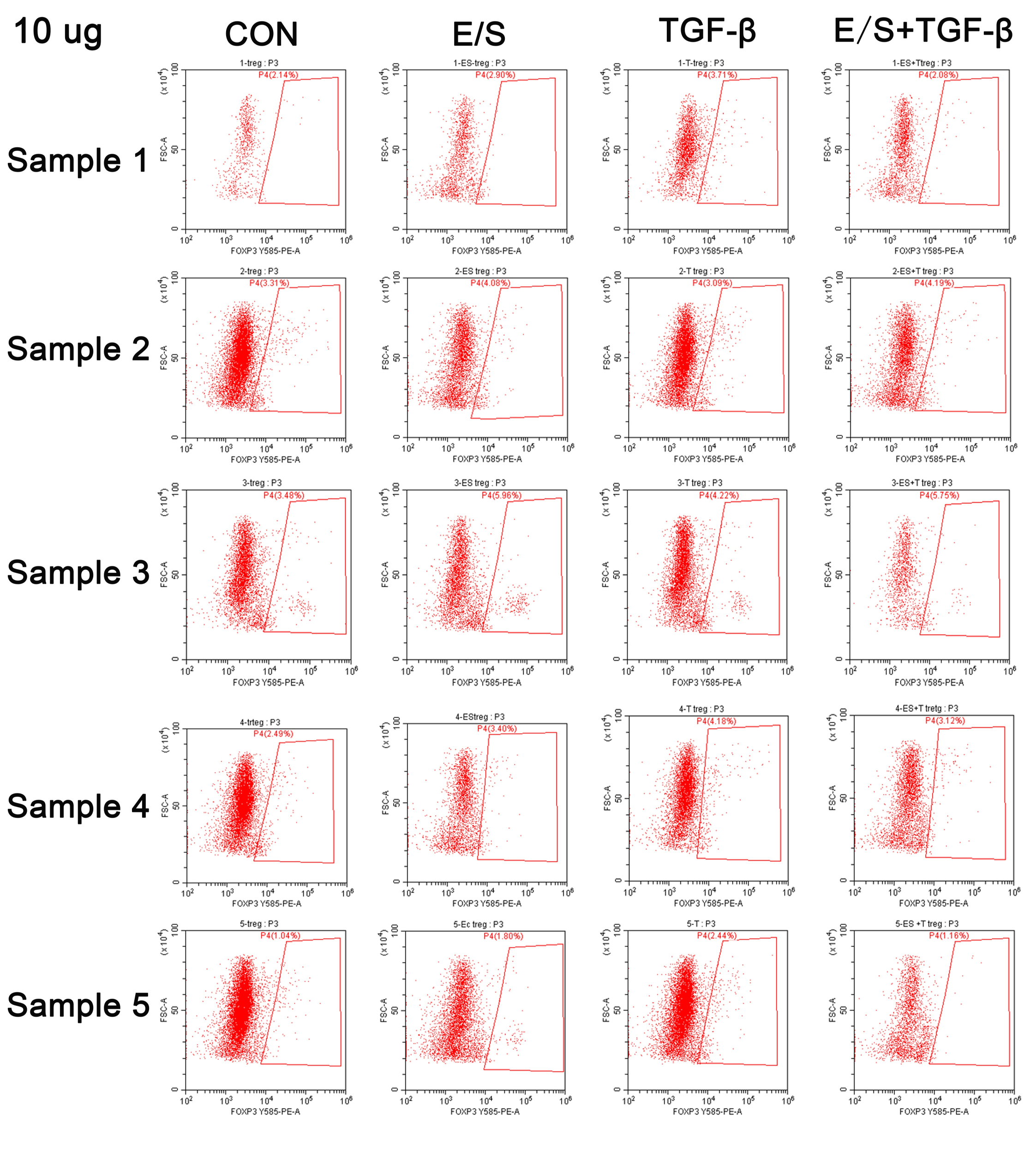

Supplement: S7 Fig — (TIF) [file pntd.0011625.s008.tif]

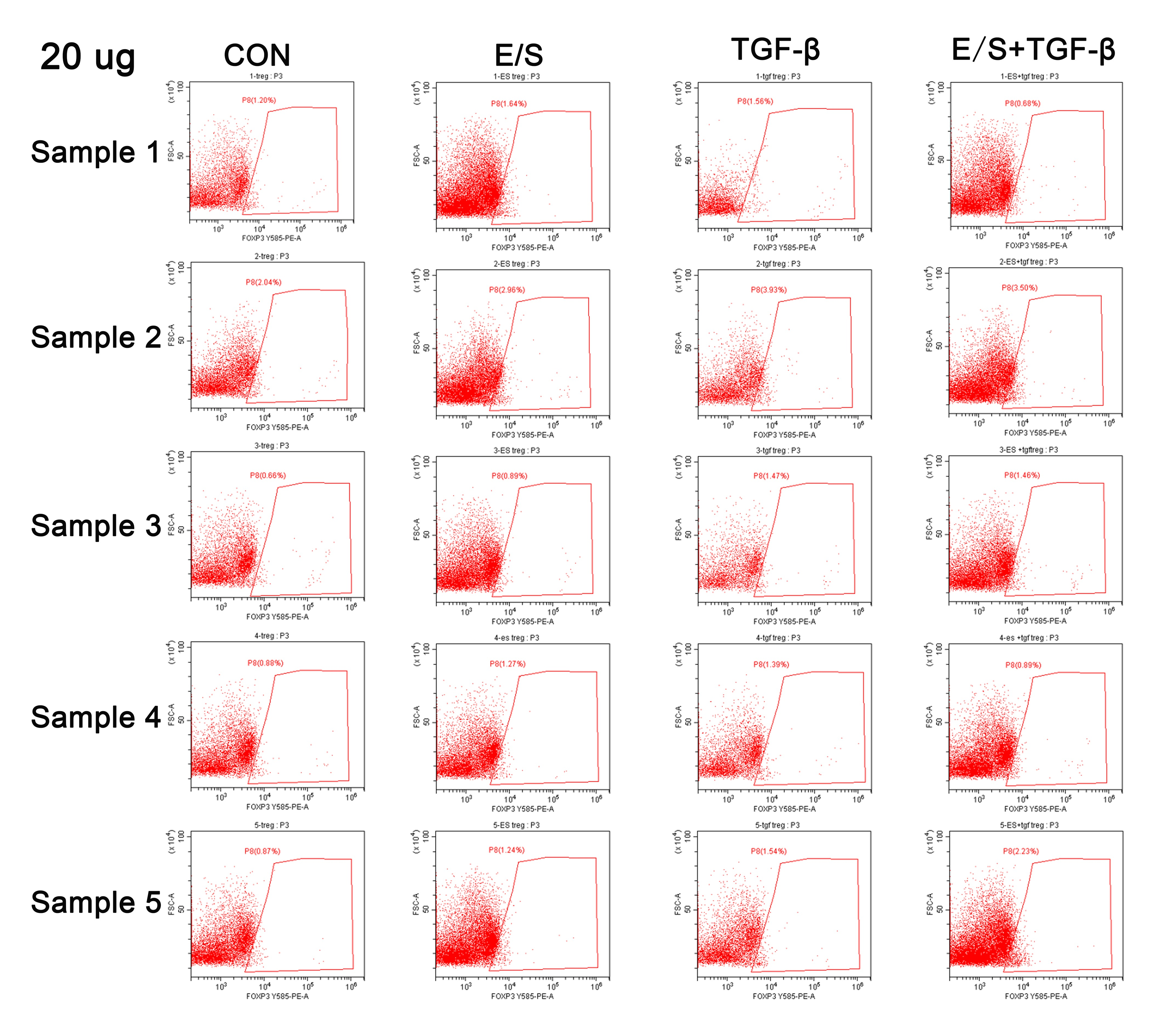

Supplement: S8 Fig — (TIF) [file pntd.0011625.s009.tif]
