# Supplementary material for: Excretory/Secretory Products from Schistosoma japonicum Eggs Alleviate Ovalbumin-Induced Allergic Airway Inflammation
Source: PLoS Negl Trop Dis. 2023 Oct 3;17(10):e0011625. doi: 10.1371/journal.pntd.0011625 (PMC10547495; doi:10.1371/journal.pntd.0011625)
Supplement: Attachment — Submitted filename: Response to Reviewers.doc [file pntd.0011625.s010.doc]

 Dear Editors,

We are submitting our revised manuscript entitled “Excretory/Secretory Products from *Schistosoma japonicum* Eggs Alleviate Ovalbumin-Induced Allergic Airway Inflammation” to the *PLOS Neglected Tropical Diseases* for your reconsideration of its suitability for publication. All authors have read and approved the manuscript. We have carefully taken the reviewers’ comments into account and provided responses to each of the points raised by the reviewers. Some necessary corrections have been made, and all the altered passages have been highlighted in yellow in revised version. Please refer to the followed our response to reviewer’s comments as below:

**Methods**

Reviewer #1: The authors clearly stated the objective of the study and have obtained permission from the appropriate ethic regulatory bodies for the study.

**Response:** Thanks for your recognition, we have carefully revised your comments as much as we can.

Reviewer #2: - It is unclear how many experiments were performed and how many mice were included in the groups or how many different spleens were used to do the in vitro experiments. Please add to the legend and show all individual mice (separate data points) from all experiments in the graphs of the figs.

- When more than two groups are included in the experiments, ANOVA should be applied with post hoc analysis between groups. This may have been applied, but this is not clearly described in the methods nor the legends. This should be adapted.
- The description of the legends for the suppl. figures is missing. Please add.
**Response:** Thanks for your suggestion, we have carefully revised above points according to your comments.

-We have added the detailed information in figure legends about the numbers of the performed experiments, mice in groups and the methods of statistical analysis used in all experiments (lines 926-936, 944-946, 956-959, 966-969, 975-979, and 983-993).

-All data were analyzed by the one-way analysis of variance (ANOVA) with Tukey test between the CON, E/S, OVA, OVA+E/S and OVA+DXM groups in our experiments, we have added the detailed information in the methods and figure legends.

-we have added the missing legends for suppl. Figures in lines 983-993.

**Results**
Reviewer #1: The results are well presented. However, the image quality and labels in the manuscript are not of quality. I kindly suggest that authors improve on this.

**Response:** Thanks for your suggestion, we have carefully improved the quality of the figures and re-upload them.

Reviewer #2: - In figure 1, the y-axis is staggered, which makes it difficult to interpret the data. Please use a continuous axis.

**Response:** Thanks for your suggestion, in figure 1, according to your comments, we revised the data of y-axis by a continuous axis. We worried that the continuous data in y-axis would result in the lower data was barely visible.

- In suppl fig 1; three displays are presented for the different concentration of ESP-SJE please combine in one graph to allow full comparison between the groups. The percentages of Treg cells seem to differ quite substantially between the three displays; this makes it hard to interpret the data and also questions the scientific rigor of the findings.

**Response:** Thanks for your suggestion, in suppl fig 1, we reanalyzed the data and combine these data in one graph to better allow full comparison between the groups. “The percentages of Treg cells seems to differ between the three displays” was due to the difference of the gate of Treg cells in CD4^+^ T cells, which may be not strict. So, we reanalyzed the data and found it didn’t show significant differ between the three different concentrations, and we upload the original data in supporting information (supporting file 1-4).

- Why does the TGFb not induce Treg cells? The positive control does not seem to be working?

**Response:** Thanks for your suggestion, in suppl fig 1, after we reanalyzed the data, TGF-β did induce the Treg cell and it show a statistical difference, which the inducible ability of Treg of E/S was almost comparable with the TGF-β. We have reupload the figure (Figure 6A) and the original data (supporting file 1-4).

- In the text is the described that also the F2 and Q4 induce more Treg cells in Suppl fig 2 (lines 412-414)- but this shows something else. These data seem to be lacking. Please add.

**Response:** Thanks for your suggestion, the results that F2 and Q4 could induce more Treg was mainly presented in Fig. 6 not Suppl fig2. We have revised the description in lines 512-515 in manuscript.

- The data in Suppl fig 1 and 2 seems very important for the message and conclusions of the paper and should be part of the main figures. If not sufficient experiments are performed to merit a main figure, please add more data to allow this.

**Response:** Thanks for your suggestion, the data in Suppl Fig 1 and 2 were very important for the conclusion and we revised and merged the data into a new Figure 6.

**Conclusions**
Reviewer #1: • The authors provided conclusions which are supported by the data presented in the manuscript.
• The limitations of the study have not been mentioned. I suggest authors provide information on the limitation of their study in the manuscript.
• The benefit of the findings to the advancement of knowledge on the topic under study have been discussed and public health relevance has also been touched on.

**Response:** Thanks for your recognition and suggestion, we have added the information on the limitation of our study in the manuscript in lines 648-660.

Reviewer #2: The data show significant, but modest changes. This is not always reflected by the accompanying text. In particular, the abstract needs to be tuned down, but also in other sections of the manuscript.
Fox example:
- line 39: ..reducing the number of eosinophils in the lungs, but not in the BAL ... (differences between different compartments)
- line 40: ...secretion of inflammatory cytokines, like ... (not all were reduced)
- line 41: ... a significant, but modest, upregulation of Treg cells ... (Differences were very modest)
- line 42-44: this conclusion is exaggerated - some of the fractions may induce a modest induction of Tregs AND several molecules were identified. But it is not tested whether those 9 indeed induce Treg cells - the way it is written now, may suggest it is. Please adapt.
- line 49-50: Also here the conclusion that inhibition of AAI is likely due to Treg cell induction is too much; this was not tested. Here only can be included that there is a modest inhibition of AAI combined with a modest upregulation of Treg cells. Whether this is linked has not been tested.
- line 52: Also the conclusion that ES/P molecules can be used for therapeutic application is too strong - at most they are likely candidates to be tested for their therapeutic application.

**Response:** Thanks for your recognition and suggestion, we have tune down these not rigorous descriptions in lines 46-47, 50, 51-52, 54, and 63-64 of the clean manuscript.

These conclusions should also be tuned down in other sections of the manuscript.

**Response:** Thanks for your suggestion, we have tune down above conclusions in other sections of manuscript including in lines 63-64 of the Abstract section, lines 86-89 of the Author Summary and lines 650-652 of the Conclusion section.

The differences in cytokine expression and the biological relevance has not been discussed in the discussion section.

**Response:** Thanks for your suggestion, we have added the description of the differences in cytokine expression and the biological relevance in the discussion in lines 489-502.

I miss an strength and limitation section in the discussion.
**Response:** Thanks for your suggestion, we have added the description of limitation and strength section in the discussion in lines 648-660.

**Editorial and Data Presentation Modifications?**
Reviewer #1: Minor Revision
**Response:** Thanks for your evaluation!

Reviewer #2: See comments above
**Response:** Thanks for your evaluation!

**Summary and General Comments**

Reviewer #1: • The authors did not present the abstract in a format recommended by the journal. I suggest the authors follow the journal recommendation and present the abstract in sections with headings
**Response:** Thanks for your suggestion, we have revised the abstract in a format recommended by the journal and added the section headings.

• There is absence of author’s summary in the manuscript as recommended by the journal. I suggest authors write on “AUTHOR’S SUMMARY” and this information should come after the “ABSTRACT”
**Response:** Thanks for your suggestion, we have added the author’s summary in the manuscript as recommended by the journal and come after the “ABSTRACT” in lines 69-95.

• Line 169: Remove the word “of” in between “with” and “ESP-SJEs”
**Response:** Thanks for your suggestion, we have removed the word “of” in between “with” and “ESP-SJEs” in line 229.

Line 169: The bracketed information “(1 mg each mouse each time)” is not clear. I suggest that this information be presented as “(1mg per mouse per time)”
**Response:** Thanks for your suggestion, we have revised the word “(1 mg each mouse each time)” into “(1mg per mouse per time)” in line 229.

• Line 256: Remove the word “using” in between “chromatography” and “with”
**Response:** Thanks for your suggestion, we have removed the word “using” in between “chromatography” and “with” in line 328.

• Line 294: Kindly provide space in between the combined word “spectrometercoupled”
**Response:** Thanks for your suggestion, it’s my mistake and we have provide space in between the combined word “spectrometercoupled” in lines 370-371.

Reviewer #2: See comments above.
**Response:** Thanks for your suggestion, we have carefully revised above comments.

Figure Files:
While revising your submission, please upload your figure files to the Preflight Analysis and Conversion Engine (PACE) digital diagnostic tool, https://pacev2.apexcovantage.com. PACE helps ensure that figures meet PLOS requirements. To use PACE, you must first register as a user. Then, login and navigate to the UPLOAD tab, where you will find detailed instructions on how to use the tool. If you encounter any issues or have any questions when using PACE, please email us at figures@plos.org.
 **Response:** Thanks for your suggestion, we have uploaded our figure files to the PACE and download the revised figures that meet PLOS requirements.
Data Requirements:
Please note that, as a condition of publication, PLOS' data policy requires that you make available all data used to draw the conclusions outlined in your manuscript. Data must be deposited in an appropriate repository, included within the body of the manuscript, or uploaded as supporting information. This includes all numerical values that were used to generate graphs, histograms etc.. For an example see here: http://www.plosbiology.org/article/info%3Adoi%2F10.1371%2Fjournal.pbio.1001908#s5.
 **Response:** Thanks for your suggestion, we have deposited all the data and detailed analysis in an appropriate repository once our manuscript to be publication.
Reproducibility:
To enhance the reproducibility of your results, we recommend that you deposit your laboratory protocols in protocols.io, where a protocol can be assigned its own identifier (DOI) such that it can be cited independently in the future. Additionally, PLOS ONE offers an option to publish peer-reviewed clinical study protocols. Read more information on sharing protocols at https://plos.org/protocols?utm_medium=editorial-email&utm_source=authorletters&utm_campaign=protocols
 **Response:** Thanks for your suggestion, we will deposite all the laboratory protocols used in our manuscript in protocols.io, once our manuscript to be publication.

We appreciate your positive consideration. Please feel free to contact me if there are any questions.

Sincerely,

Wei Hu

School of Life Sciences, Fudan University,

Shanghai 200438, China
